# FlowKit: A Python Toolkit for Integrated Manual and Automated Cytometry Analysis Workflows

**DOI:** 10.3389/fimmu.2021.768541

**Published:** 2021-11-05

**Authors:** Scott White, John Quinn, Jennifer Enzor, Janet Staats, Sarah M. Mosier, James Almarode, Thomas N. Denny, Kent J. Weinhold, Guido Ferrari, Cliburn Chan

**Affiliations:** ^1^ Duke Center for AIDS Research, Duke University, Durham, NC, United States; ^2^ Department of Biostatistics and Bioinformatics, Duke University Medical Center, Durham, NC, United States; ^3^ Center for Human Systems Immunology, Duke University Medical Center, Durham, NC, United States; ^4^ BD Life Sciences - FlowJo, Ashland, OR, United States; ^5^ Duke Immune Profiling Core, Duke University School of Medicine, Durham, NC, United States; ^6^ Department of Surgery, Duke University Medical Center, Durham, NC, United States; ^7^ Duke Human Vaccine Institute, Durham, NC, United States

**Keywords:** systems immunology, flow cytometry, software, python (programming language), single cell data science, FlowJo, GatingML

## Abstract

An important challenge for primary or secondary analysis of cytometry data is how to facilitate productive collaboration between domain and quantitative experts. Domain experts in cytometry laboratories and core facilities increasingly recognize the need for automated workflows in the face of increasing data complexity, but by and large, still conduct all analysis using traditional applications, predominantly FlowJo. To a large extent, this cuts domain experts off from the rapidly growing library of Single Cell Data Science algorithms available, curtailing the potential contributions of these experts to the validation and interpretation of results. To address this challenge, we developed FlowKit, a Gating-ML 2.0-compliant Python package that can read and write FCS files and FlowJo workspaces. We present examples of the use of FlowKit for constructing reporting and analysis workflows, including round-tripping results to and from FlowJo for joint analysis by both domain and quantitative experts.

## Introduction

Despite the phenomenal advances in Single Cell Data Science (SCDS) methodology and an ever-growing collection of algorithms and open-source packages, it is an open secret that the day-to-day analysis of cytometric data in flow laboratories and core facilities is still predominantly performed using traditional software, especially FlowJo. There are good reasons for this - traditional software such as FlowJo excels at the visual manipulation and analysis of data, and human analysis is inherently more adaptable than any fully automated workflow. For example, domain experts are typically better at removing debris, dead cells, and cell aggregates by gating than automated approaches. However, there are also severe limitations to a purely manual workflow for data analysis, especially the poor scalability to high-volume workflows and limitations of visual discovery for high-dimensional data sets.

We developed FlowKit to bridge the gap between manual and automated workflows. Specifically, we wanted to develop a robust basis for foundational cytometry operations, provide a straightforward interface to SCDS algorithms, and facilitate the integration of manual and automated analysis. To ensure that foundational cytometry operations are supported, we checked for full compliance with Gating-ML 2.0 (1), hence ensuring that compensation, transformation, and gating operations were all implemented correctly. To interface with data science, machine learning and computer vision algorithms, we developed FlowKit so that analytic results and event data could be exported as a generic pandas DataFrame, a standard unit for analysis in Python scientific workflows and interoperable with the R, Spark, and SQL frameworks. To allow integrative manual and automated analysis, we worked with FlowJo developers to ensure that FlowKit could read *and* write FCS and FlowJo workspace files, allowing the round-tripping of data and analytic results to and from FlowJo.

We chose Python as the implementation language for pragmatic reasons - Python is the dominant language for data science, machine learning and computer vision; the two main deep learning frameworks, TensorFlow and Torch, use Python as their *de facto* language; Python is now often taught as a first programming language in many quantitative disciplines; and Python has a robust ecosystem for scalable workflows and system integration. While R has a rich set of libraries for cytometric data analysis, we believe that FlowKit provides a complementary alternative with access to state-of-the-art data science and machine learning frameworks for Python developers and will be welcomed by the cytometry bioinformatics community. We anticipate and hope that the Python bioinformatics community will build advanced tools for quality control (QC), analysis, visualization, and even graphical user interfaces (GUI) on top of the foundational features provided by FlowKit.

## Methods

### Data Sets

The data used in this manuscript were generated by the External Quality Assurance Program Oversight Laboratory (EQAPOL) Flow Cytometry Program. The program is designed to assess the proficiency of NIH/NIAID/DAIDS-supported and potentially other research laboratories interested in performing Intracellular Cytokine Staining (ICS) assays. A detailed account of the methods used to generate the data is provided in a previous publication (2).

### Organization of FlowKit

The structure of FlowKit is simple, with only two building block classes: the *Sample* class for handling individual Flow Cytometry Standard (FCS) samples, and the GatingStrategy class for compensation, transformation, and gating operations. A third Session class is used for coordinating complex analytic workflows with multiple FCS samples and gating strategies. For standard analytic pipelines, these three classes suffice.

**Sample**. The Sample class represents a single sample and is typically created by reading in an FCS file acquired from either a flow or mass cytometer. It provides methods to retrieve the data and metadata, methods to sub-sample, filter, compensate, transform, and visualize sample events. Event data can be exported as a pandas DataFrame, with options to export the unprocessed, compensated, or transformed events. Compensation is designed as an optional step for compatibility with pre-compensated flow cytometry data sets or FCS files acquired *via* mass cytometry. Sample instances can also be created from a NumPy array or a pandas DataFrame, which can easily be exported to a new FCS file.

**GatingStrategy**. The GatingStrategy class represents a hierarchical gating strategy that includes instructions for compensation and transformation. Compensation matrices can be read in from multiple sources or created *de novo* from a set of compensation bead files. The *GatingStrategy* class supports the linear, log, channel ratio, inverse hyperbolic sine (asinh), hyperlog, logicle and FlowJo biexponential transformations, as well as Boolean, quadrant, rectangular, elliptical, and polygon gates. Instances of this class can be edited to programmatically customize hierarchical gating strategies.

**Session**. A Session represents a collection of gating strategies and FCS samples. FCS samples are added and assigned to sample groups, and each sample group has a single gating strategy template. The gates in a template can be customized per sample to accommodate variations cell sub-populations due to data acquisition. In practice, a *Session* instance is often populated from a FlowJo workspace file and its associated FCS files, though a FlowJo workspace is not required, and a Session can be completely constructed programmatically.

### Support for FCS Files

FlowKit reads FCS 2.0, 3.0 and 3.1 files and writes FCS 3.1 files. Care has been taken to ensure that FlowKit can read common FCS files generated by diverse cytometers - typically contributed as bug reports from FlowKit users - including files that contain variable integer sizes and files that violate the standards specification with an off-by-one error. FlowKit also correctly interprets and applies both the gain and time step values from the FCS metadata.

### Display of Transformed Events

FlowKit labels plot axes with the transformed values to be as transparent as possible regarding the display of processed data. While most flow cytometry analysts are familiar with scatter plots from FlowJo where the transformed data is presented with axes displaying the non-transformed event values, in a programmatic context this approach becomes problematic. Since FlowKit allows the retrieval of processed event data (compensated, transformed, and/or gated), an end user of FlowKit would receive exported data in the transformed space. If the user then went on to programmatically create a gate based on those events, the gate definition (e.g., coordinates of polygon vertices) would be in the transformed space. Displaying the transformed data values in FlowKit generated plots eliminates the need for end users to transpose gate boundary locations when using those plots as a guide for defining gates.

### Unit Tests and Coverage

FlowKit currently has a suite of over 270 unit tests covering 91% of the code base. These tests include all the GatingML-2.0 compliance tests. To run these tests, issue the following commands in the top level of the FlowKit repository:

*python setup.py build_ext –inplace*

*python run_tests.py*

### Dependencies

FlowKit’s dependencies are listed on the GitHub page, and include FlowIO and FlowUtils, also developed by our group. FlowIO is a pure Python package for reading and writing FCS files with no external dependencies, while *FlowUtils* handles low-level compensation and transformation tasks. *FlowKit* and *FlowUtils* use C extensions to improve performance.

### License and Installation

FlowKit uses the open-source and permissive BSD 3-Clause License and is freely available from https://github.com/whitews/FlowKit. It supports Python 3.6-3.9 and can be installed using *pip install flowkit*.

### Maintenance and Support

The development effort for FlowKit is funded by the Duke Center for AIDS Research (CFAR), the Duke Center for Human Systems Immunology, and the External Quality Assurance Program Oversight Laboratory (EQAPOL). The core features for processing of FCS and workspace files are complete, and future releases will focus on improving and maintaining compatibility with FlowJo and any emerging FCM standards, as well as optimization and bug fixes. Inquiries or issues regarding the use of FlowKit can be submitted to the issues section of the GitHub website (https://github.com/whitews/FlowKit/issues).

## Results

### Features and Software Comparison

While R has a rich set of libraries for cytometric data analysis *via* the Bioconductor ecosystem, the Python community lacks a counterpart with feature parity to the foundational flow cytometry libraries found in Bioconductor (specifically the flowCore (3) and flowWorkspace (4) packages). We developed FlowKit as an alternative to the R ecosystem (**Table 1**), to provide bioinformaticians using Python for cytometric analysis a reliable foundation to build upon. To that end, FlowKit is designed to facilitate accurate pre-processing of FCS data, including proper interpretation of event data using FCS metadata, adherence to gate definitions in the GatingML 2.0 specification, and convenient extraction of processed data for use in external libraries. Features available in FlowKit include:

1. Read/Write FCS Files
1.1. Read FCS files, supporting FCS versions 2.0, 3.0, and 3.1
1.2. Proper application of cytometer channel gain and timestep information from FCS metadata
1.3. Export FCS data as new FCS files, NumPy arrays, pandas DataFrame objects, and CSV files
2. Compensation
2.1. Compensate events using spillover matrices from various sources (FCS metadata or exported FlowJo compensation file)
2.2. Create a compensation matrix from a set of compensation bead files
3. Transformation
3.1. Support for a variety of transformations used in the flow community, including logicle, inverse hyperbolic sine (asinh), and FlowJo-compatible bi-exponential transforms
4. Gating
4.1. Full support for the GatingML 2.0 specification
4.2. Limited support for importing FlowJo 10 workspace files, including importing and exporting FlowJo 10 WSP files
4.3. Programmatically create gating strategies including polygon, rectangle, range, ellipsoid, quadrant, and boolean gates
4.4. Easily retrieve gating results from a gating strategy as a pandas DataFrame
5. Visualization
5.1. Histogram of single channel data
5.2. Contour density plot and interactive scatter plot of two channels
5.3. Interactive scatter plot matrix of any combination of channels
5.4. Interactive scatter plots of gates with sample events

**Table 1:** Comparison of features between FlowKit and other FCM software libraries.

	FlowKit	flowCore (R)	Cytoflow	CytoPy	FlowCal	FlowCytometryTools
Version	0.8.0	2.4.0	1.1.1	2.0.1	1.3.0	0.5.1
(release date)	(Oct 2021)	(Nov 2020)	(Mar 2021)	(May 20221)	(Jan 2021)	(Jan 2021)
Programming Language	Python	R	Python	Python	Python	Python
(latest version supported)	(3.9)	(4.1)	(3.7^2^)	(3.9)	(3.8)	(2.7, 3^5^)
Uses Continuous Integration	Yes	Yes	No	No	No	Yes
Includes tests	Yes	Yes	Yes	Yes	Yes	Yes
Reports test coverage	Yes	No	No	No	No	No
FCS file library	FlowIO	*built-in*	fcsparser^3^	FlowIO	*built-in*	fcsparser
Supports channel gain ($PnG)	Yes	Yes, but not by default	No	No	Yes, but not by default	No
Supports $TIMESTEP	Yes	No	No	No	No	No
Supports logicle	Yes	Yes	Yes	Yes (via FlowUtils)	Yes^4^	No
Supports FlowJo bi-exponential	Yes	Yes	No	No	No	No
Supports hierarchical gating	Yes	Yes^1^	No	Yes	No	No^6^
Supports GatingML 2.0	Yes	Yes	No	No	No	No
Supports FlowJo 10 workspaces	Yes	Yes^1^	No	No	No	No

1. flowCore does not include hierarchical gating or FlowJo WSP support directly, this functionality is in the related flowWorkspace library.

2. Cytoflow is installable through Anaconda.

3. Cytoflow uses an internal fork of fcsparser.

4. The FlowCal logicle function is implemented in Python as opposed to C for the other libraries in this list.

5. It is unclear which Python versions are supported, PyPI states 2.7, the README file states “python 3”.

6. FlowCytometryTools supports a CompositeGate class to combine gates but does not have an API to explicitly create gate hierarchies.

### Use Cases

**Figure 1:**
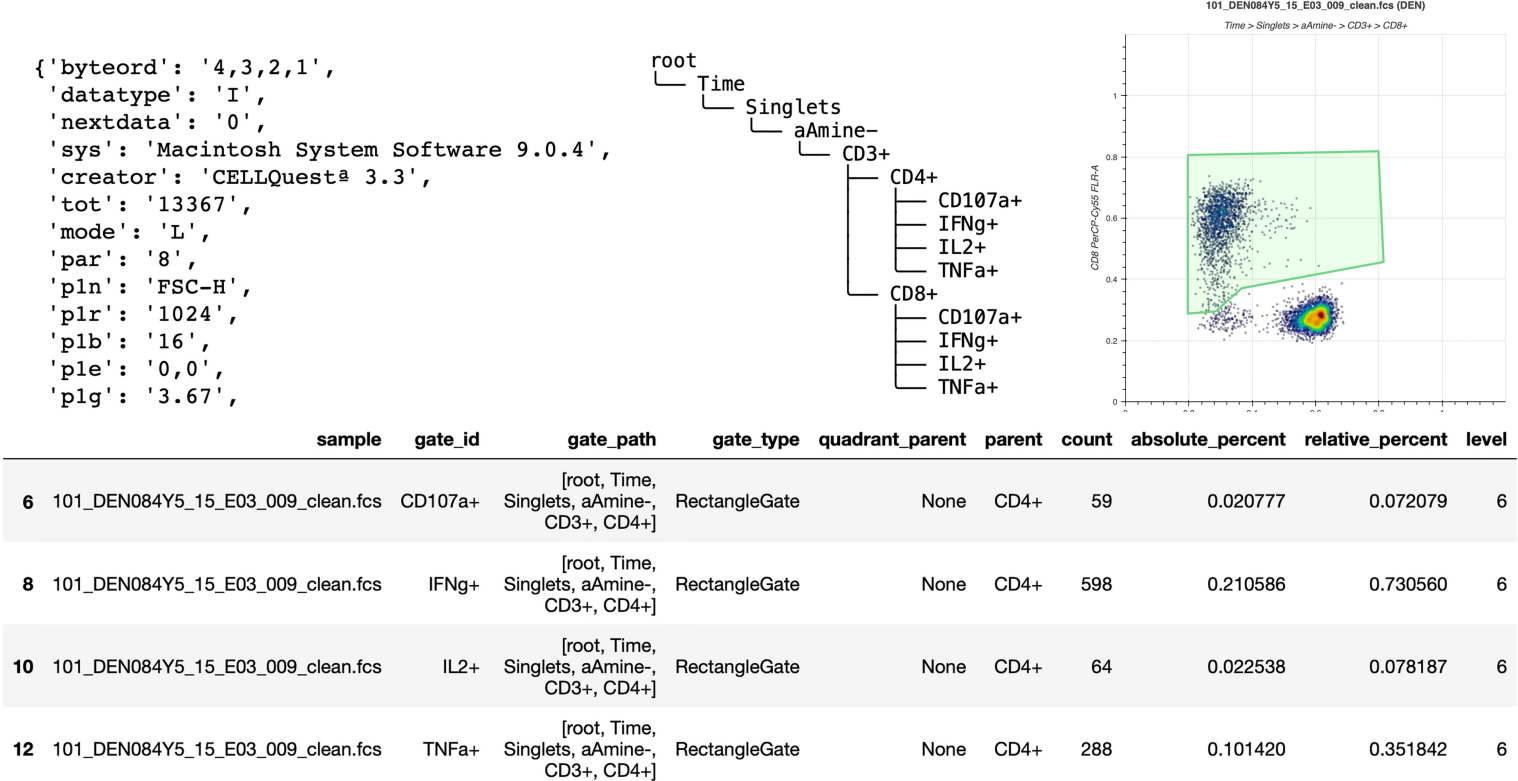
Basic elements provided by FlowKit for generating reports and downstream analysis. Clockwise from top left – metadata as key:value pairs, gating hierarchy as ASCII text, scatter plot of a gate, DataFrame of results from applying gating strategy specified in the Session class.

**Figure 2:**
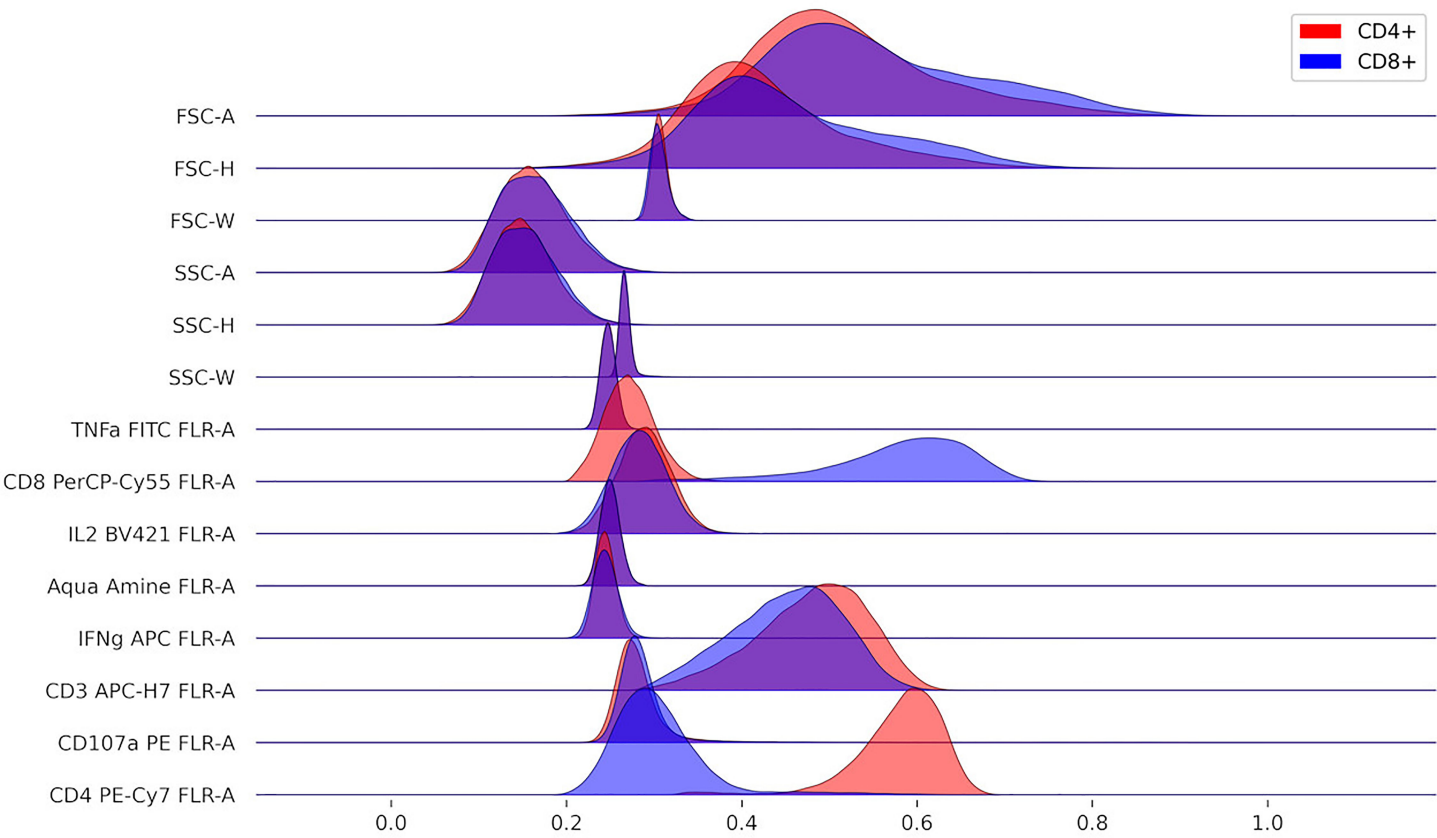
Comparison of marker distributions across CD4+ and CD8+ T cells.

**Figure 3:**
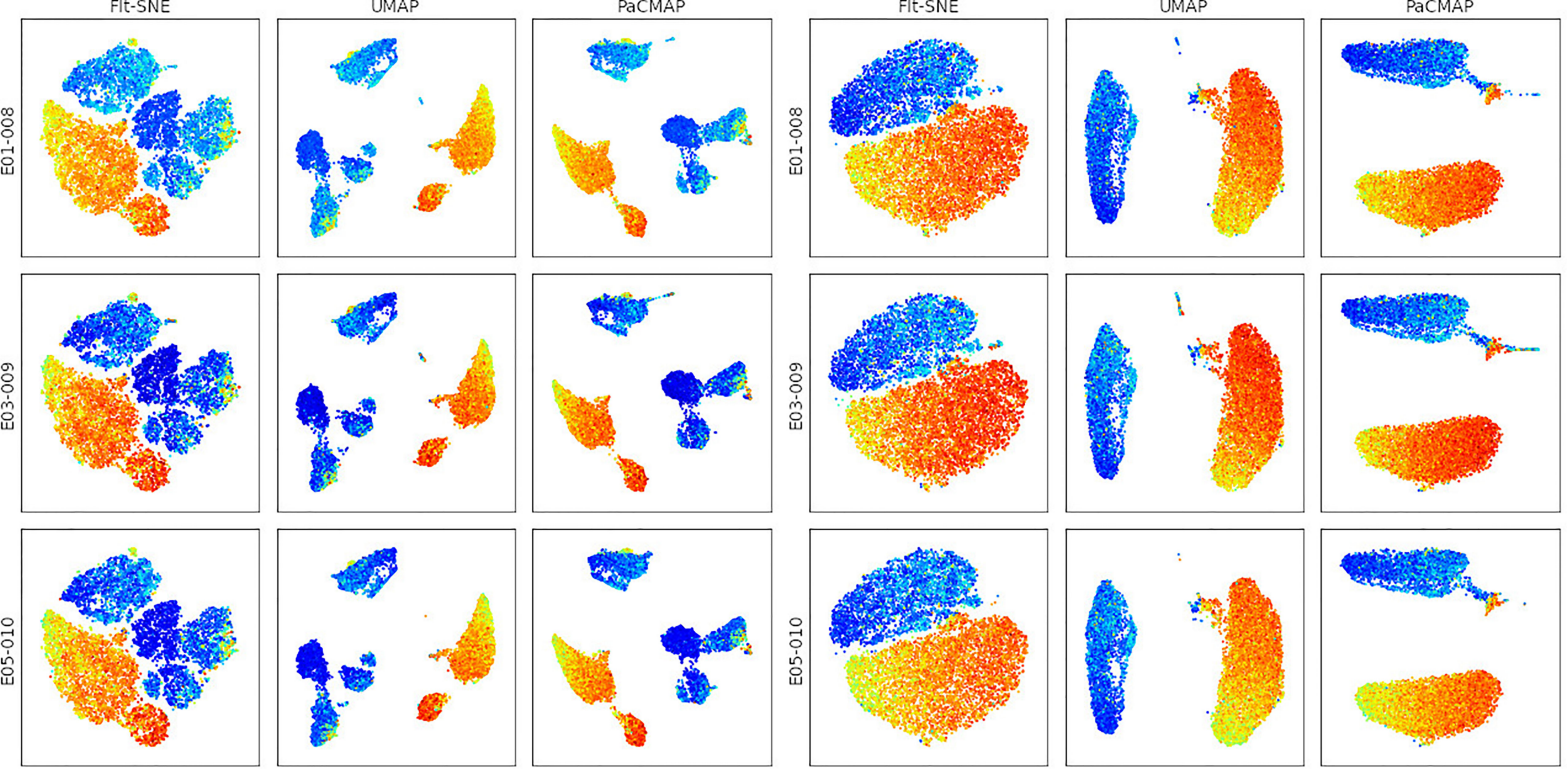
Comparison of dimension reduction algorithms on the Singlets (left) and CD3+ (right) gated events. Events are pseudo-colored by the CD4 marker intensity.

**Figure 4:**
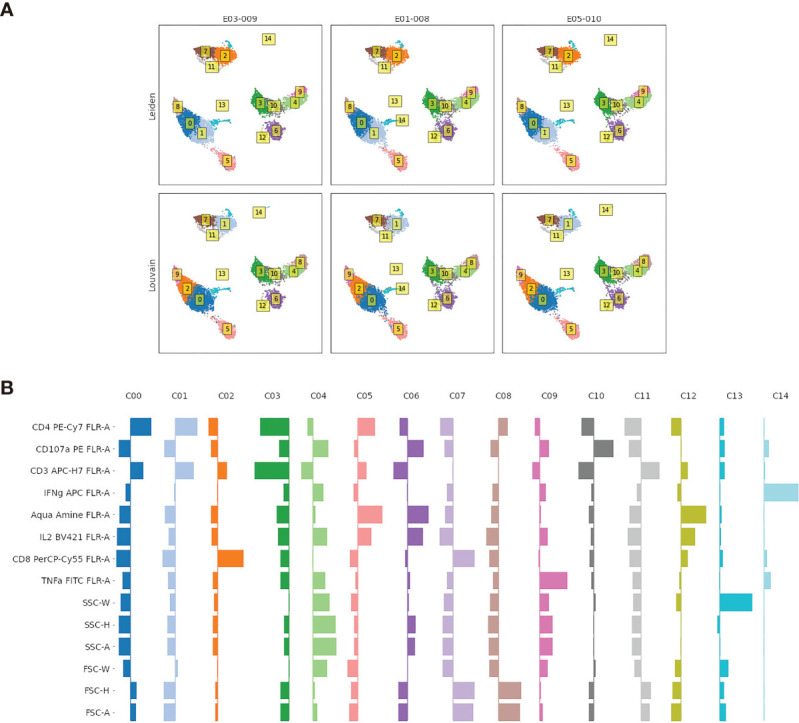
**(A)** Comparison of the Leiden and Louvain community detection algorithms for clustering flow cytometry data. Labels in boxes are the assigned cluster indexes. Events plotted using PaCMAP for dimension reduction and colored according to the event cluster label. **(B)** Visualization of marker distribution for each cluster found using the Leiden algorithm shown in the top panel. Distributions are for values scaled to have zero mean and unit standard deviation; values to the left (right) of the thin vertical indicate mean marker values for that cluster that are below (above) the average for all cells.

**Figure 5:**
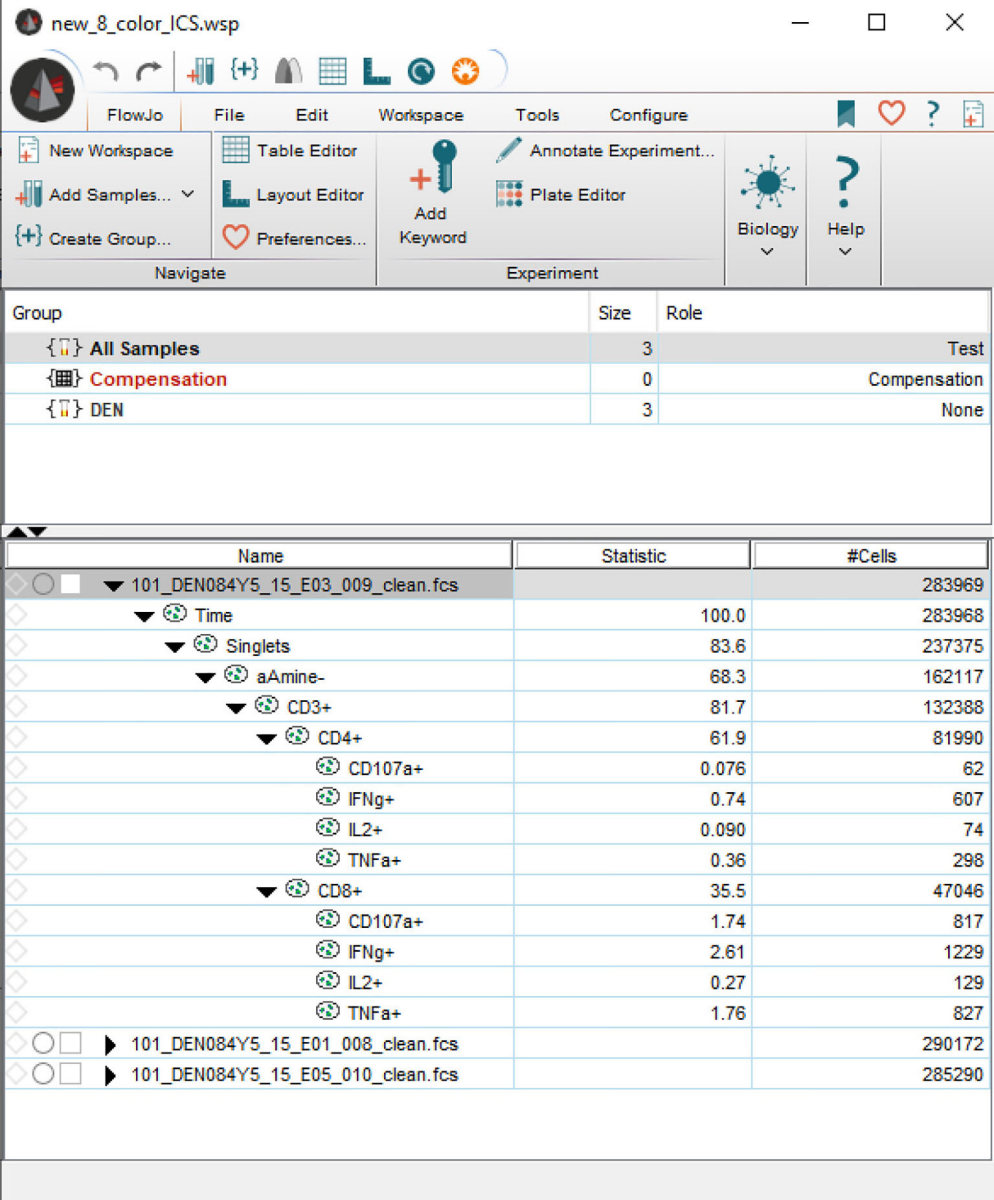
Screenshot taken from FlowJo, showing an imported workspace that is entirely programmatically constructed.

We illustrate the application of FlowKit with four common use cases. While FlowKit can be used independently of FlowJo, we present use cases where a Session is initiated with a FlowJo workspace file and its associated FCS files to illustrate integrative manual and automated analysis. All examples including those that do not use FlowJo workspace files can be found in the GitHub repository in the examples folder. All examples and use cases are provided as Jupyter notebooks:

- **Figures 1**, **5** are from examples/flowkit-session-replicate-flowjo-wsp.ipynb
- **Figures 2**, **3** are from examples/dimension_reduction_on_gated_populations.ipynb
- **Figure 4** is from examples/clustering_comparison_leiden_vs_louvain.ipynb

#### Generating a Report From a FlowJo Workspace

One of the most basic tasks is to generate a report about a sample group that has been analyzed in FlowJo. While FlowJo can obviously export gated results, doing so *via* code is more flexible and powerful. For example, it is simple to *join* the gated data with results from a separate complete blood count (CBC) assay to calculate the absolute cell counts (cells per unit volume of blood) for each gated subset. **Figure 1** shows some of the basic elements directly provided by FlowKit to generate such reports, including metadata key:value pairs, the gating strategy, several options to visualize individual gates, and a table of gating results.

Since FlowKit also provides access to the compensated, transformed, and gated events, more advanced reports can provide informative summaries of the event data in each gate. A simple example is shown in **Figure 2**, where ridgeline plots of each marker distribution are constructed to compare events from the CD4+ and CD8+ gates.

#### Creating SCDS/Machine Learning Workflows

Of course, having programmatic access to the gated events makes it simple to develop automated analysis workflows that begin with events from a specific gate using other tools or libraries outside of FlowKit. This provides the opportunity to integrate manual analysis to generate filtered basic cell subsets (i.e., remove debris, aggregates, dead cells, and gate well-known cell subsets such as T and B lymphocytes) with automated analysis to explore these subsets in depth. We illustrate the ease of extending FlowKit with the common SCDS operations of dimension reduction, clustering based on community detection algorithms, and pseudo-coloring by arbitrary functions of the markers.

In **Figure 3**, we show the application of three different dimension reduction methods on events from two different gates (‘Singlets’ and ‘CD3+’) to the 3 samples in the sample group. The three dimension reduction methods are FFT-accelerated Interpolation-based t-SNE (FIt-SNE) (5), Uniform Manifold Approximation and Projection (UMAP) (6), and Pairwise Controlled Manifold Approximation (PaCMAP) (7). Note that the PaCMAP algorithm is not available within FlowJo, showing that FlowKit makes it easy to apply and evaluate cutting-edge SCDS algorithms.

In **Figure 4A**, we evaluate two clustering methods, the Leiden (8) and Louvain (9) algorithms, that identify *modular* communities in graph-based representations of the event data. These algorithms are popular in the scRNA-seq community as they are fast and generate biologically plausible cluster labels, and appear to generate reasonable cluster structures in our example.

In **Figure 4B**, we explore the marker distributions of the clusters generated by the Leiden algorithm (top panel in **Figure 4A**). Such visualizations can be used to aid the interpretation of individual clusters as distinct cell types by domain experts. As explained in the next section, the cluster labels can be added to an augmented FCS file, and automatically derived gates around each cluster added to the gating strategy for thorough review by a domain expert in FlowJo.

#### Generating Output for FlowJo (“Round-Tripping”)

In this last example, we load a FlowJo workspace and then programmatically reconstruct the workspace from the ground up in a FlowKit session. During the reconstruction, we programmatically add a sample group, compensation matrix, transforms and multiple types of gates. Finally, we export the reconstructed workspace as a FlowJo WSP file and read it in using FlowJo. **Figure 5** shows the reconstructed workspace as viewed in FlowJo. In practice, this will allow information from automated analysis (e.g., addition of additional feature columns such as dimension-reduced coordinates or illustrating a discovered cell cluster with ellipsoidal gates constructed from the cluster covariance) to be reviewed in a familiar setting by domain experts. Here we pseudo-colored events in the dimension-reduced plots by the fluorescence intensity of the CD4 marker, but it would be trivial to use any other function for coloring (e.g., CD4:CD8 ratio).

## Discussion

We present FlowKit to the cytometry bioinformatics community as a software construction kit that is capable of large-scale secondary analysis of data in repositories such as ImmPort (10, 11). FlowKit is most similar in functionality to the R package flowCore (with flowWorkspace), especially in the support for the full range of common transformations, hierarchical gating, GatingML 2.0, and FlowJo workspaces. One feature available in FlowKit but not flowCore is the ability to export programmatically constructed or modified gating hierarchies to FlowJo WSP files. There are four other Python packages that have functional overlap with FlowKit and show evidence of recent development activity, namely Cytoflow (12), CytoPy (13), FlowCal (14), and FlowCytometryTools (15), but none of them have feature parity with flowCore. We also note that one of the major Python packages, CytoPy, builds on top of our low-level packages FlowIO and FlowUtils. In short, FlowKit is unique among Python FCM packages in providing a feature-complete programmatic interface to FCM data and FlowJo workspaces, allowing the FCM community to leverage the vast Python statistics, data science and machine learning ecosystem for automated analysis or the creation of new software tools. In this manuscript and in notebooks available in the FlowKit GitHub repository, we provide examples of creating workflows that integrate manual and automated analysis and can take advantage of the latest SCDS algorithms being [s1] developed.

FlowJo support is limited to FlowJo 10 WSP workspace files. For use with FlowKit, FlowJo generated JO files need to be imported into a recent version of FlowJo (>10.8) and exported as WSP files. In principle, it is possible to use GatingML-2.0 files to specify gating strategies, and FlowKit supports the import and export of GatingML-2.0-compliant files, opening a route to FlowKit by non-FlowJo users. In practice, we are not aware of any laboratory or software that routinely generates and uses GatingML-2.0 files, probably because of the inconvenience of working at an individual sample level. The current version of FlowKit also does not support importing and exporting WSP files with custom modifications to individual samples within a sample group; this is due to a potentially ambiguous interpretation of such strategies when gates with the same name at different levels are used. We are working with FlowJo developers to remedy this and expect this functionality to be implemented soon.

We emphasize that FlowKit is a toolkit that is meant to be built upon; it is most similar in purpose to the combination of the flowCore (3) and flowWorkspace (4) packages in R/Bioconductor. One design difference from flowCore is that we favor the ubiquitous DataFrame over more specialized formats to facilitate interoperability with other data science libraries in Python. For example, the pandas DataFrame is compatible with analysis using TensorFlow or Spark, facilitating the use of accelerated and distributed processing of cytometry data. Where necessary, it is simple to wrap the DataFrame in a richer data structure, for example, AnnData for compatibility with scanpy (16). Inspired by the ecosystem that has grown around flowCore and flowWorkspace, we hope that the Python bioinformatics community too will build an ecosystem of cytometry quality control, alignment, visualization, and analysis packages on it.

## Data Availability Statement

Publicly available datasets were analyzed in this study. This data can be found here: https://github.com/whitews/FlowKit/tree/0.8.1/examples.

## Author Contributions

CC directed the project. SW is the software developer of the FlowKit library. JQ and JA provided assistance and information to implement compatibility with the FlowJo application. JS, JE, and SM provided domain expertise on manual flow cytometry analysis. GF, KW, and TD provided immunological domain expertise. SW and CC contributed equally to the writing of the manuscript with consultation from all the authors. All authors contributed to the article and approved the submitted version.

## Funding

This research is supported in part through an EQAPOL collaboration with federal funds from the National Institute of Allergy and Infectious Diseases, National Institutes of Health, Contract Number HHSN272201700061C, and by the Duke University Center for AIDS Research (CFAR), a National Institutes of Health (NIH) funded program (5P30 AI064518).

## Conflict of Interest

Authors JQ and JA were employed by BD Biosciences - FlowJo.

The remaining authors declare that the research was conducted in the absence of any commercial or financial relationships that could be construed as a potential conflict of interest.

## Publisher’s Note

All claims expressed in this article are solely those of the authors and do not necessarily represent those of their affiliated organizations, or those of the publisher, the editors and the reviewers. Any product that may be evaluated in this article, or claim that may be made by its manufacturer, is not guaranteed or endorsed by the publisher.
